# A pilot study integrating environmental simulation for climate responsive architecture and planning curricula

**DOI:** 10.1007/s44217-026-02391-z

**Published:** 2026-11-14

**Authors:** Ying Zheng, Malachy Buck, Tom Jefferies, Saul Golden, Clare Mulholland, Nuala Flood

**Affiliations:** 1 School of Natural and Built Environment, Queen’s University Belfast, Belfast, Northern Ireland UK; 2 Belfast School of Architecture and the Built Environment, Ulster University Belfast, Northern Ireland, UK

**Keywords:** Environmental simulation, Sustainable design education, Rhino and grasshopper, Ladybug tools, Architectural and planning education

## Abstract

This paper extends pedagogic research on the use of environmental digital simulation in higher education by examining how digital tools within an architecture and planning studio can impact knowledge, skills, practices and professional attitudes towards climate-responsive design. This study developed as part of Arts and Humanities Research Council (AHRC) Future Island Island (FII) project that applied wider cross-disciplinary, community and industry engaged research toward enhancing the green transition of sustainable design practices and education in Northern Ireland. This study presented centres on a series of teaching workshops through which third-year (final year) architecture and planning undergrade students engaged with Rhino, Grasshopper and Ladybug Tools to interrogate environmental analysis. Applying their learning both cohorts subsequently translated these analyses into decisions for urban planning and building design. Drawing on eight semi-structured interviews, the findings indicate: (1) participants perceived increased confidence and a developing ability to interpret environmental simulation outputs; (2) persistent barriers, including hardware limitations, software installation challenges and the cognitive demands of visual programming; and (3) a marked and seemingly durable enhancement in sustainability literacy, accompanied by a stated intention to integrate simulation into future design practice. As an initial proof-of-concept, the study provides preliminary evidence concerning the feasibility of a studio-embedded environmental simulation teaching approach across Architecture and Planning contexts. This pilot suggests that supporting students’ engagement with climate-responsive environmental simulation requires pre-configured digital environments, scaffolded learning pathways and coordinated teaching across Architecture and Planning.

**Supplementary Information:**

The online version contains supplementary material available at https://doi.org/10.1007/s44217-026-02391-z.

## Introduction

Education is key to improving people’s climate literacy and understanding (UN, [53]; Kumar, [24]). Within the scope of a UKRI funded Future Island-Island (FII) project in Northern Ireland, part of the Arts and Humanities Research Council (AHRC)’s Future Observatory: Design the Green Transition programme. This study presents education-focused and professional practice-informed research “to create engaging learning materials and experiences that connect people of all ages with their environment, empowering them to shape its future” Future Island-Island (2024) [16]. The paper draws on FII’s Theory of Change, discussed below as an innovative approach to design-led research to frame a focused pedagogic examination of how embedding digital environmental simulation tools within architecture and planning education can improve climate literacy, understanding and a greater depth of professional skill longer term. Beyond shaping attitudes and behaviours, formal education in architecture and planning can equip learners’ generations with the knowledge and skills to recognise environmental issues and to evaluate how adaptive design strategies perform against real-world contexts. Meanwhile, the Education for Sustainable Development for 2030 (ESD for 2030) roadmap urges higher education not only to convey sustainability knowledge to students but to enable action ([28]; UNESCO, [52]).

Within Architecture, RIBA’s Themes and Values for Architectural Education now underpin validation in the UK and globally ([20]; [29]). RIBA Education and Professional Development Framework place a new emphasis on those areas of expertise and experience that impact the quality and performance of the built environment such as prioritising end-user protection, climate literacy and responsible specification [22]. This prioritises the health wellbeing of those who use buildings and spaces, as well as our wider duties to society and the natural environment [9]. The framework directs curricula towards the emerging Mandatory Competences, addressing climate change and building safety, promoting ethical practice, and developing business and research skills [22]. While preserving the architecture field’s commitment to innovative, visually articulate design, it re-elevates the technical agenda, calling for renewed scrutiny from visiting boards and a proportionate response in the work of students and educators.

Similarly, within planning, the Royal Town Planning Institute (RTPI) positions climate action as a strategic priority for the profession and education [8]. RTPI’s 2020–2030 strategy foregrounds climate action, and its joint guidance with the Town and Country Planning Association (TCPA) frames climate change as the top priority for planning practice (TCPA and RTPI, 2020 [50]). The Policy Statement on Initial Planning Education specifies the knowledge, skills and values that accredited degrees must deliver, with application subsequently tested through the Assessment of Professional Competence (APC) and maintained via the core continuing professional development (CPD) framework, including emphasis on digital and climate capabilities (RTPI, 2019 [46]). RTPI also provides practice advice and tools for plan-making and development management (e.g., the Planning for Climate Change directory), reinforcing the expectation that climate literacy and evidence-informed decision-making are baseline outcomes for initial planning education (RTPI, 2019 [46]).

Despite this strong policy direction, significant gaps remain. Although sustainability has been embedded in built-environment curricula for decades, the urgency and complexity of the climate crisis expose continuing shortcomings in mainstream provision ([32]; Schiano-Phan and Gonçalves, [47]). Sustainability topics in planning and architecture programmes are often dispersed within isolated modules, rather than embedded as a cohesive and central framework throughout the curriculum [19, 38]. Within both subject, there is a tendency to silo education away from built environment disciplines, such as architecture, landscape, and environmental science, which limits the development of transdisciplinary solutions to complex climate and urban resilience challenges [31]. There is also a lack of hands-on training in simulation tools and spatial analytics that can bridge the gap between policy concepts and applied environmental design.

As a result, a more robust transdisciplinary approach is urgently required, one that informs and empowers planning and architecture students through alternative pedagogical paradigms, enhanced digital tools, and more diverse skillsets. Existing research documents curriculum reforms and the integration of digital tools as well as a growing body of literatures that emphasises simulation tool functionality, performance evaluation, workflow reviews and case studies showing how performance simulation can support early design decision-making.

For example, several researchers have proposed a diversity of methods to incorporate sustainability into higher education or into university curricula by proposing new courses, models for evaluation or manuals on how to integrate sustainable education and curricula, while others advocate for systemic restructuring of universities to address this challenge [27]. One notable example is the work of Ceulemans and De Prins [11], who developed a comprehensive teacher’s manual and a four-step integration method as part of the HET IVOOR project in Belgium. This initiative aimed to support the incorporation of sustainable development (SD) and corporate social responsibility (CSR) across higher education curricula. The manual combined conceptual foundations with ready-to-use, student-centred teaching resources and was designed to assist both pre-service and in-service educators from various disciplines. Notably, its horizontal integration approach empowers individual educators to embed sustainability into their own courses, even in contexts where institutional backing was limited. Within the context, a research question: “Is it feasible and acceptable to embed environmental simulation knowledge and workflows into Architecture and Planning teaching through a studio-embedded workshop?”

This is a pilot study because it prioritised assessing the feasibility and acceptability of embedding an environmental simulation-led workflow within existing studio timetables across two institutions. The pilot study objectives are to: (1) explore how students with varying levels of self-reported prior digital experience engaged with and completed the core activities of a five-week workshop; (2) identify infrastructural and cognitive barriers that might undermine equitable participation; and (3) generate preliminary evidence on how students translate environmental metrics into decision making, consistent with the Future Island Island (FII) Theory of Change. Accordingly, the scope of this study is limited to examining the feasibility and perceived pedagogical value of a five-week, supplementary environmental simulation workshop, including students’ reported experiences, early conceptual understanding, perceived application of environmental information to design and planning decisions, and barriers to participation. The study does not assess objective software competency, causal learning gains, longer-term retention, or comparative differences between Architecture and Planning students.

## Digital tools, design and environmental education

Beyond curriculum-focused reforms, other scholars have emphasised the transformative role of digital tools in planning and design education [49]. Representative studies include early stage, simulation supported decision tools and workflows [3, 51], reviews of simulation-based optimisation for building performance [13, 18, 35], urban scale energy modelling syntheses (Reinhart and Cerezo, [41]), and dynamic daylight metrics for design (Reinhart and Cerezo, [41]).

Given the growing importance of such tools across the design, planning and development sectors, there is a need to develop the student’s skills, and understanding of such tools with existing planning and architecture programs (Cazacove and Semiali, 2025; [10]). A challenge is that within both disciplines there is often a tendency to teach such tools in isolation, rather than as a central part of the design process (Kalpkirmaz and Voss, 2020; [31]).

To address this, several studies have examined the integration of such tools within integrate design education (e.g. in design studio teaching). This work centres on the role of building performance simulations in design, and how such tools could be utilised to enhance environmental outcomes within the built environment. Several studies found that use of different simulation tools enhanced understanding of the relationship between building design choices and energy use, enhanced motivation for learning through increased interaction (Cazacove and Semiali, 2025; [7]). Rizaoglu and Voss, [44] undertook multi-institutional surveys and identified that such tools might be most effective when introduced early in the design process, make use simplified simulation tools, close supervision and feedback from tutors.

Despite this, much of the existing work examines education within disciplinary silos, despite much of the skills inherent within environmental simulation tools being shared across the built environment disciplines [42]. Opoku and Guthrie, [37] and Korobar and Siljanoska [23] both argue that breaking down these silos and instead developing connections between theory and application between disciplines is essential for effective sustainability education. Therefore, in this spirit this study seeks to examine how simulation tools not only develop digital skills to enhance sustainability education but also examines simulation as a pedagogical mechanism to bridge across architecture and planning. This will occur within studio-embedded teaching, with this environment been identified as a setting that promotes interactivity and learning by doing (Neuman, [34]; [31]).

It does so through reflecting upon a semester-long, co-taught pilot delivered across Queen’s University Belfast and Ulster University. A curated toolkit (Rhino, Grasshopper and Ladybug Tools) within existing programmes to scaffold a pathway from basic climate literacy (e.g., EPW interpretation) to performance-informed design moves (e.g., solar exposure and outdoor thermal comfort mapping). The pilot formed part of Future Island-Island (FII; Grant Ref: AH/Y003780/1) and the UK-wide Future Observatory: Design the Green Transition programme. Prior to this study, neither program has implemented formal teaching experiments employing environmental simulation as an integrative pedagogical tool, nor had established a collaborative cross-institutional approach to advance this dimension of built-environment education and future-skills enhancement.

Digital tools used in built-environment education vary considerably in complexity, functionality and licensing models (AI-Nusair et al., 2025; [12, 54]). They range from commercial platforms such as Adobe Creative Suite, AutoCAD, DesignBuilder, ENVI-met and Autodesk Insight, to specialised systems including Building Information Modelling (BIM) and Geographic Information Systems (GIS) [4]. While some provide free versions with limited functionality or discounted educational licenses, the majority still require paid subscriptions or institutional agreements, posing practical barriers to sustained use in academic settings, particularly in a climate of financial constraints within higher education (QAA, [40]). More critically, although these tools provide powerful analytical capabilities, each is typically designed to serve specific domains of environmental analysis, such as energy consumption, microclimate modelling, or visual communication, making it difficult to adopt a unified language to describe what the tools could do [3]. This fragmentation not only hinders interdisciplinary understanding but also creates challenges for educators aiming to integrate these tools meaningfully across studio-based and technical modules. In addition, many of these platforms demand high computational power and hardware specifications, which further limit their accessibility for students and institutions with constrained resources.

Environmental simulation inherently demands interdisciplinary thinking, combining aspects of spatial design, environmental science, and data-driven analysis. Thus, it provides a valuable pedagogical pathway for enhancing students’ capacity to incorporate climate-responsive strategies into both architectural and planning workflows. The toolkit developed in this study thus serves not only as a technical solution but also as means promote more integrated and holistic design thinking among learners [2-3]. By combining environmental simulation into the core of architectural and planning education, students across disciplines can be better prepared to address spatial and climatic challenges of future sustainable development.

## Method and materials

**Fig. 1:**
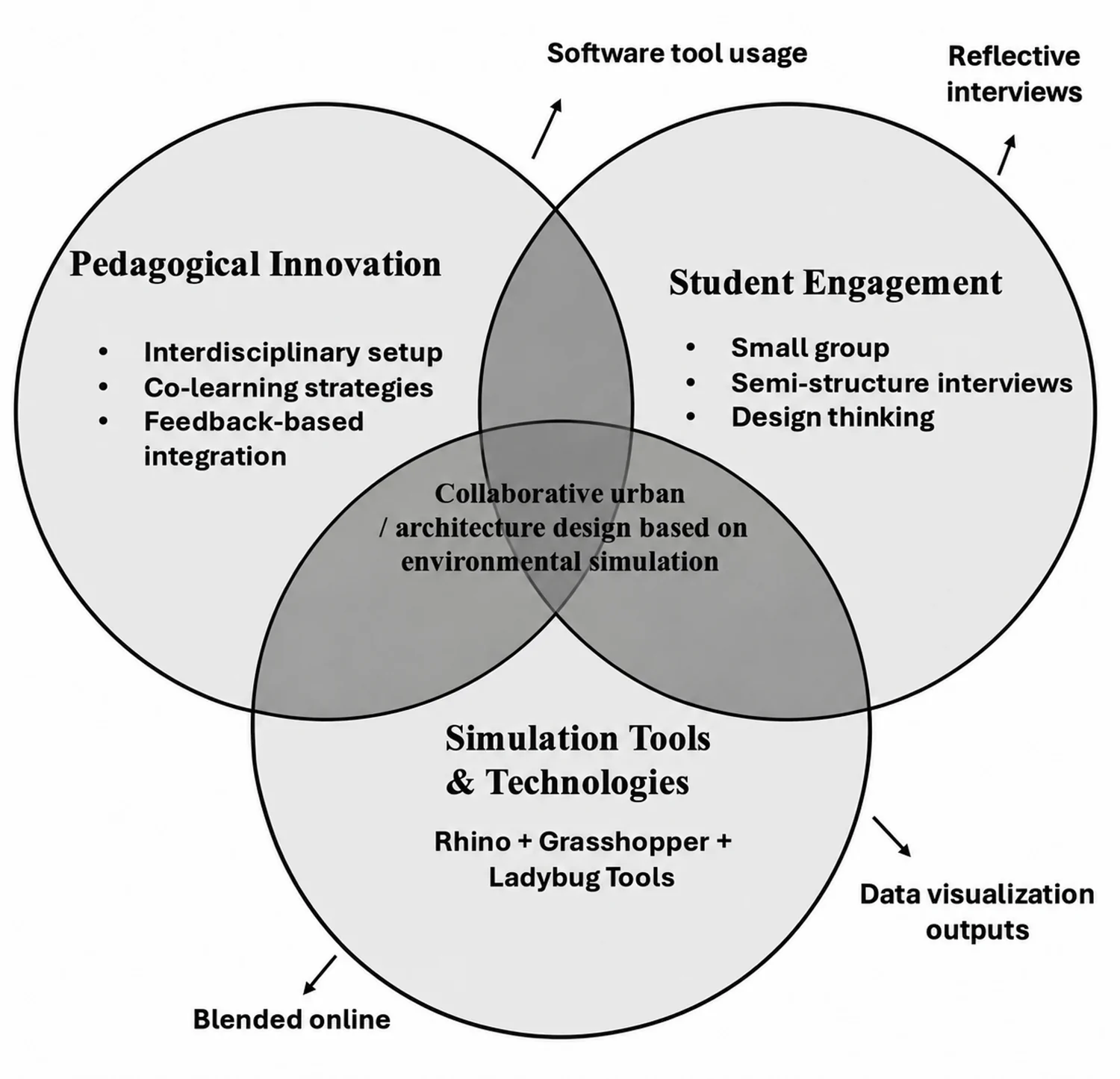
The conceptual framework of the study, illustrating the intersection of pedagogical innovation, student engagement strategies, and environmental simulation technologies in a collaborative learning environment (Author’s own)

This study situates a studio embedded environmental simulation workshop within architectural and planning simultaneously within both QUB and Ulster University, specifically targeting curriculum developments that support sustainable design and environmental analysis. As illustrated in Fig. 1, the research adopts an integrated framework that synthesises three core dimensions: pedagogical innovation, student engagement, and simulation tools & technologies. This tripartite model is designed to examine the efficacy of cross-disciplinary collaboration, specifically between undergraduate programmes at Queen’s University Belfast (Architecture) and Ulster University (Planning), in co-designing and visualising sustainable futures. Accordingly, the study was designed as a pilot to assess feasibility and acceptability across two institutional timetables and to refine the teaching pathway and evaluation instruments prior to a larger-scale study.

This pilot study is positioned within an interpretivist paradigm, focusing on participants’ sense-making of simulation-led learning within studio contexts. Data sources comprised (i) semi-structured interviews (primary) and (ii) teaching observation during the workshop (secondary), including the exercise of daylighting, solar radiation and UTCI maps, and written reflections embedded in studio submissions.

Within the proposed framework (see Fig. 1, left), Pedagogical Innovation and Simulation Tools & Technologies define the intervention architecture, while Student Engagement functions as the evaluation lens through which the intervention is assessed. Specifically, the pedagogical dimension shows how the workshop was designed and delivered, with an interdisciplinary set-up, co-learning strategies, and iterative feedback integration that scaffolded students’ movement from deeper understanding of climate-related challenge to design decisions. The tools-and-technologies dimension specifies the enabling infrastructure that operationalised this pedagogy in a design-proximal manner, using a shared workflow (Rhino, Grasshopper, and Ladybug Tools) to generate interpretable climate evidence (e.g., daylighting hours, solar radiation and UTCI mapping) that could be brought into studio or planning discussions. This method allowed for a critical reflection on how simulation is applied and perceived across the two disciplines.

To evaluate the student engagement component (see Fig. 1, right). In this pilot study, all 13 participants were invited to participate in the semi-structure interviews. Eight students agreed to participate, while five declined. The interview sample comprised seven planning students and one architecture student. The cohort was evenly split by gender, with four male and four female participants. All students participated voluntarily on an anonymous and informed-consent basis. Participants were recruited from two universities and two disciplinary contexts; however, the interview sample was predominantly composed of planning students.

The interviews explored students’ prior familiarity with simulation tools, perceived learning outcomes, challenges encountered, and reflections on applicability to future design scenarios. Each interview lasted approximately 10–15 min and followed a semi-structured protocol guided by five questions (listed below), providing consistency while permitting probing of individual experiences [39]. Semi-structured interviews were selected for their interactive, conversational qualities that mirror real-world exchanges between researchers and participants [14, 39], thereby providing rich feedback in person delivery modes utilized in the workshop. All interviews were audio-recorded with participant consent and subsequently transcribed. This method allowed for a critical reflection on how simulation is applied and perceived.

Interview questions (open-ended):

Q1. How has your understanding of environmental simulation evolved during the workshop?

Q2. What were the main challenges you faced while learning and applying environmental simulation in your project?

Q3. How did this studio shape your understanding of sustainable design and co-design processes?

Q4. How did the workshop structure, assignments, or teaching methods impact your learning experience? Were there any teaching practices you found particularly effective or in need of improvement?

Q5. How did you engage with the environmental simulation tools (e.g., Rhino, Grasshopper, Ladybug Tools) throughout the workshop?

Transcripts were analysed using a codebook thematic analysis. Two researchers independently open-coded an initial subset (~ 25%) to align a preliminary codebook; one researcher then coded the remainder with periodic peer debriefs, and discrepancies were discussed until interpretive stability. Both researchers were involved in delivering the workshop. This dual instructor–researcher role provided contextual understanding of the teaching process but can also have influenced participants’ interview responses. Participants have been inclined to provide favourable accounts because of the existing instructor student relationship. Voluntary participation, anonymisation and the use of open-ended questions were intended to reduce this risk. This positionality and the associated potential for social desirability bias were therefore considered when interpreting the findings. Finally, all six researchers and staff review all the contents. Themes were organised at two levels: (i) learning gains (climate literacy, tool confidence, translation to design decisions) and (ii) enablers/barriers (installation friction, hardware bottlenecks, cognitive load/pacing, support formats). Teaching artefacts were used for triangulation (evidence of analysis layers; presence/quality of decision pathways).

Ethical approval was granted by the Queen’s University Belfast Faculty Research Ethics Committee (Faculty REC; Reference: EPS 25_16) within the Future Island-Island research framework overseen by Ulster University. The institutional application ID is Application QUB v1_20250120. All participants provided written informed consent for audio recording and the anonymised use of interview excerpts and teaching artefacts. Data were stored on secure university servers and handled in accordance with institutional GDPR policies.

### Future Island–Island (FII) and the theory of change

Future Island–Island (FII; AH/Y003780/1) is a UKRI-funded programme within the Future Observatory: Design the Green Transition initiative. Within the FII Green Transition Ecosystem, the Architects of Change (AoC) programme provides a relevant pedagogical reference point by operationalising sustainability learning as agency-building, design-led, and practice-based [21]. AoC positions participants as future “change-makers” and explicitly addresses the tension between short-term actions and long-term green transition goals through workshop formats grounded in design-thinking [21]. Its reported learnings emphasise learning-by-doing, hands-on engagement, and learner autonomy, alongside mixed-method evaluation using post-workshop surveys, creative artefacts, and researcher reflections to strengthen credibility [21]. Building on these principles, while working with a different learner group and modality, our study adopts a studio-embedded simulation workflow that uses rapid visual feedback to support evidence-informed design or planning reasoning and captures proximal outcomes through interviews. Architects of Change provided a practice-based pedagogical reference point through its emphasis on hands-on sustainability learning, learner engagement and environmental agency; it was not treated as a formal or validated theory. The FII Theory of Change was used as a programme-level logic model to inform the design focus of this pilot study. In this pilot study, the ToC informed three specific aspects of the research design: (1) the selection of analysis tools with rapid visual feedback to support evidence-to-decision translation; (2) the five-week workshop that progressed from basic climate literacy to applied interpretation; and (3) the evaluation focus on how students narrated and justified “design moves” using simulation outputs, rather than on software application.

### Setting and design

The environmental simulation workshop was delivered as a five-week, studio-embedded pilot under the Future Island–Island (FII) Green Transition programme. The FII research team brings together researchers from Queen’s University Belfast (Architecture) and Ulster University (Planning), and this cross-institutional collaboration was translated into a joint teaching intervention linking a third year (final year) Architecture studio at QUB and Planning module at UU. A five-week format was adopted to balance undergraduate timetabling with the cognitive load of introducing simulation to mixed-background cohorts, enabling scaffolded progression from foundational climate literacy to be applied interpretation while maintaining continuity with concurrent studio and planning coursework. The five week pilot program is summarised below in Table 1, with a more detailed description and explanation of each week content in the following section.

**Table 1:** Summary of five-week teaching plan

Week 1	Introduction to Environmental Simulation	• Introduced pilot program and Rhino/Grasshopper and Ladybug Tools.
Week 2	Annual Climate and Wind Rose Analysis	• Verified EPW climate data • Produced 2D/3D climate and wind-rose analyses using a Belfast case study.
Week 3	Daylight Hours Analysis and Solar Radiation	• Generated sunlight-hours and solar-radiation maps in Ladybug • Ran orientation sensitivity tests
Week 4	Thermal Comfort Analysis by Universal Thermal Climate Index	• Introduced UTCI as an outdoor thermal comfort index • Produced comfort maps across seasons and times
Week 5	Review and Q&A	• Review and Q&A • Discussed applying tools to studio projects

The weekly cadence supported ‘low-floor’ or ‘high-ceiling’ tasks so that all learners achieved core milestones, with optional extensions for advanced exploration, improving equity of outcomes across heterogeneous prior experience. Each session combined a short concept briefing, a guided digital practical and a translated analysis into performance-informed design moves. All students were expected to complete the core analytical milestones (climate data visualisation, daylight-hours and solar-radiation mapping; UTCI outdoor thermal comfort mapping). Discipline-specific extensions were then introduced to support relevance and equity: Architecture students were encouraged to translate outputs into early-stage building surrounding analysis, whereas Planning students were encouraged to translate outputs into site appraisal narratives and spatial decision-making. This structure avoided allocating tasks strictly “by expertise” while still enabling differentiated, profession-relevant application.

A 3D visualisation driven workflow enabled through Rhino 8, Grasshopper and Ladybug Tools was selected because it aligns with early-stage design pedagogy by enabling rapid, parametric ‘what-if’ exploration that links design moves to quantitative feedback. It provides an open, extensible modelling, evaluation environment with strong interoperability for cross-disciplinary use in architecture and planning (Rhino3D, 2025; Grasshopper, [17]; Ladybug Tools, [26]). This workflow keeps modelling and environmental analysis in a single contiguous space, reducing cognitive and logistical overhead for undergraduates. Besides, it ensures reproducibility and traceability of assumptions through explicit parameterisation and versionable scripts [33]. Furthermore, it sustains an accessible entry level for beginners yet remains scalable for advanced research in the future.

The teaching pathway introduced key principles of climate-responsive design and progressed through EPW-based climate literacy (including wind roses and the representativeness of typical meteorological years), cumulative solar radiation and sunlight-hours analysis, and sketch-level Universal Thermal Climate Index (UTCI) mapping using Rhino & Grasshopper with Ladybug Tools [45]. Emphasis was placed on understanding, practice and applying outputs to early design decisions, such as, situation analysis or microclimate mitigation. This workshop focuses soley on simplified early-stage environmental analysis, excluding detailed Computational fluid dynamics (CFD) or whole building energy modelling. The intended learning outcomes were to develop working knowledge of Ladybug-based environmental analysis techniques, to demonstrate practical use of climate data for sustainability and green-transition questions, and to enable defensible, evidence-based early design decisions grounded in simulation outputs.

#### Session 1: Introduction to environmental simulation

**Fig. 2:**
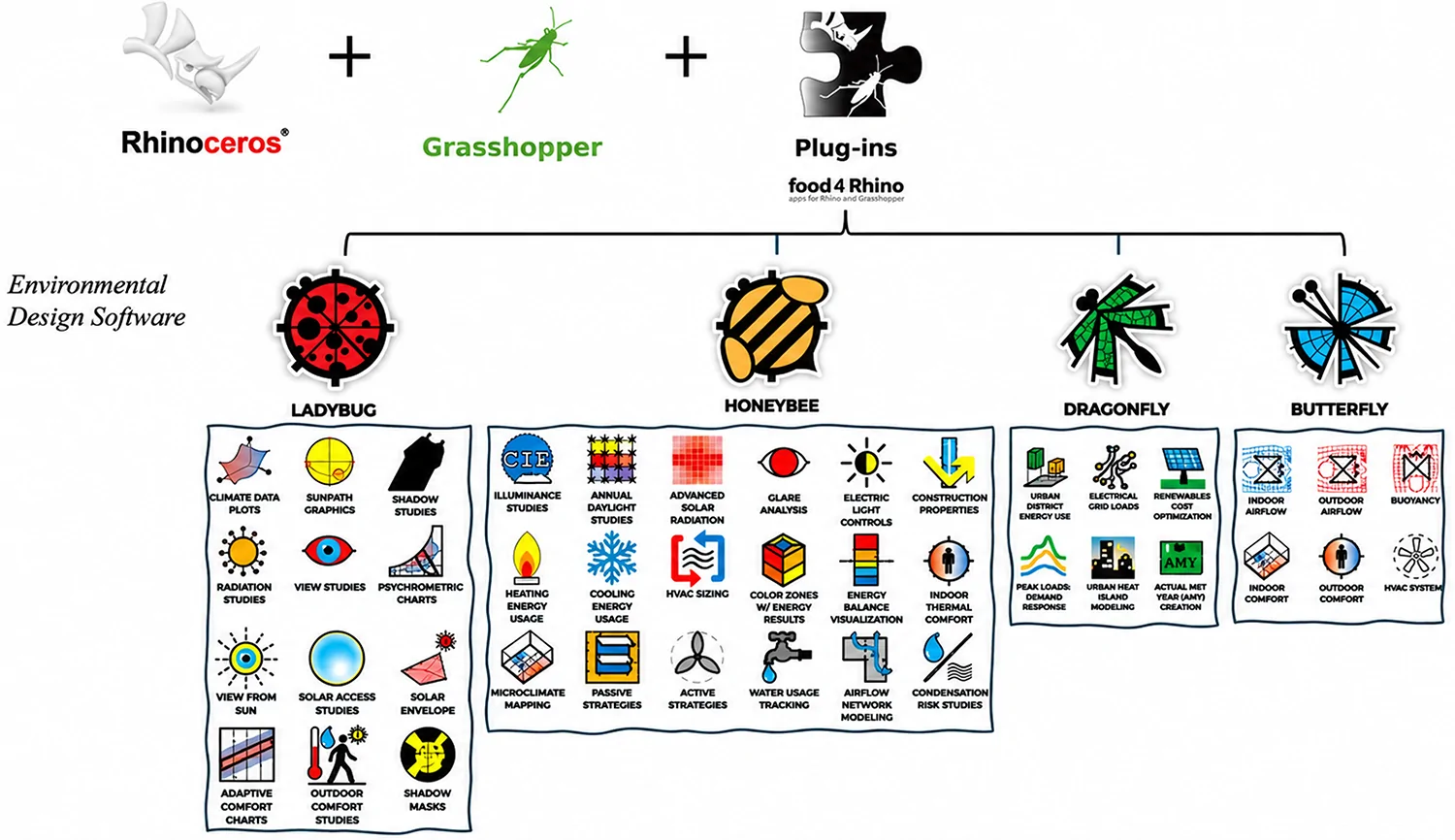
Environmental workflows (author’s own)

The first session introduced Rhino 8 and Grasshopper, establishing the foundational parametric-modelling knowledge required for subsequent environmental analyses. Rhino 8, Grasshopper provides a widely adopted Non-Uniform Rational B-Spline NURBS model and mesh-based environment for architecture, engineering, fabrication and construction, supporting associated geometries, constraint-driven exploration and rapid iteration across early design stages (Rhino3D, 2025 [43]). Base site and massing geometries were generated in Grasshopper and used as hosts for environmental workflows (see Fig. 2).

A guided overview of Ladybug Tools followed. Ladybug, designed for integration with parametric environments, enables early, design-proximal climate analysis, including EPW parsing, cumulative solar radiation and sunlight-hours mapping, and outdoor thermal comfort (UTCI) interpretation suitable for early decision-making (Ladybug Tools, [26]). Interoperability with companion plugins (Ladybug, Honeybee, Butterfly and Dragonfly) was noted to increase awareness of various environmental simulation approaches and to situate Ladybug within the broader analytical ecosystem. The emphasis of the education pilot remained on Ladybug due to its suitability for early-stage workflows. Teaching delivery to EPW-based climate literacy, solar radiation, sunlight hours and UTCI mapping within Ladybug, with no CFD or detailed energy modelling workflows were executed in this education pilot.

To minimise installation friction, a standardised teaching pack and the newest versions were provided. Supporting engines required by the ecosystem (e.g., EnergyPlus and Radiance/OpenStudio) were pre-installed to ensure compatibility with downstream analyses. Within this five-session block, delivery was limited to Ladybug-based EPW literacy, solar/sunlight diagnostics and UTCI; no CFD or detailed energy modelling was executed [5]. OpenFOAM (CFD) and the Butterfly plugin were present on some lab images for completeness but were not used in teaching (OpenFOAM, 2025 & Ladybug Tools, 2025 [36]). This delineation ensured that the session focused on evidence-informed, early-stage environmental reasoning rather than engineering-level simulation [3].

#### Session 2: Annual climate and wind rose analysis

**Fig. 3:**
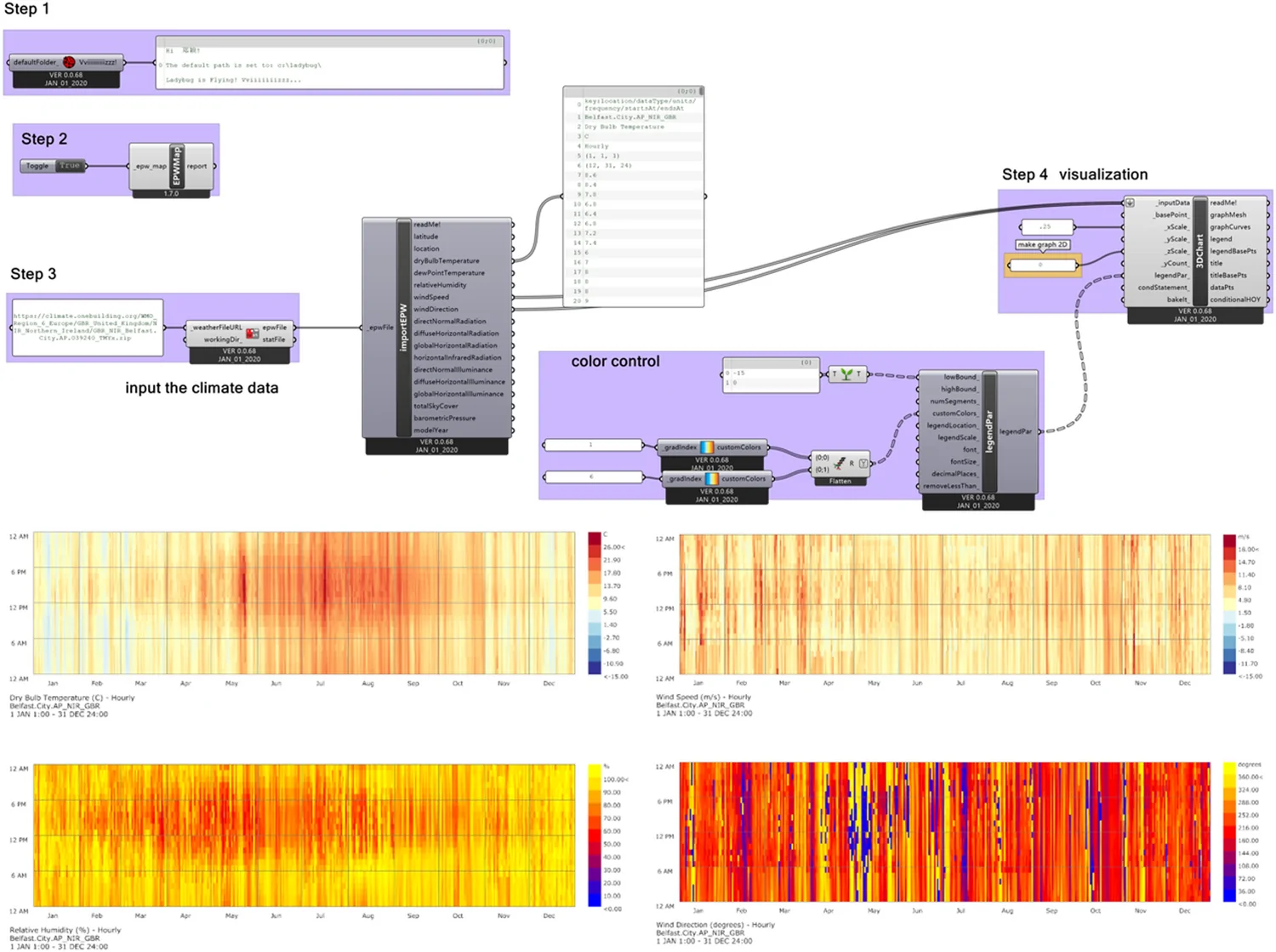
Customise weather data visualisation with 2D/3D chart (author’s own)

**Fig. 4:**
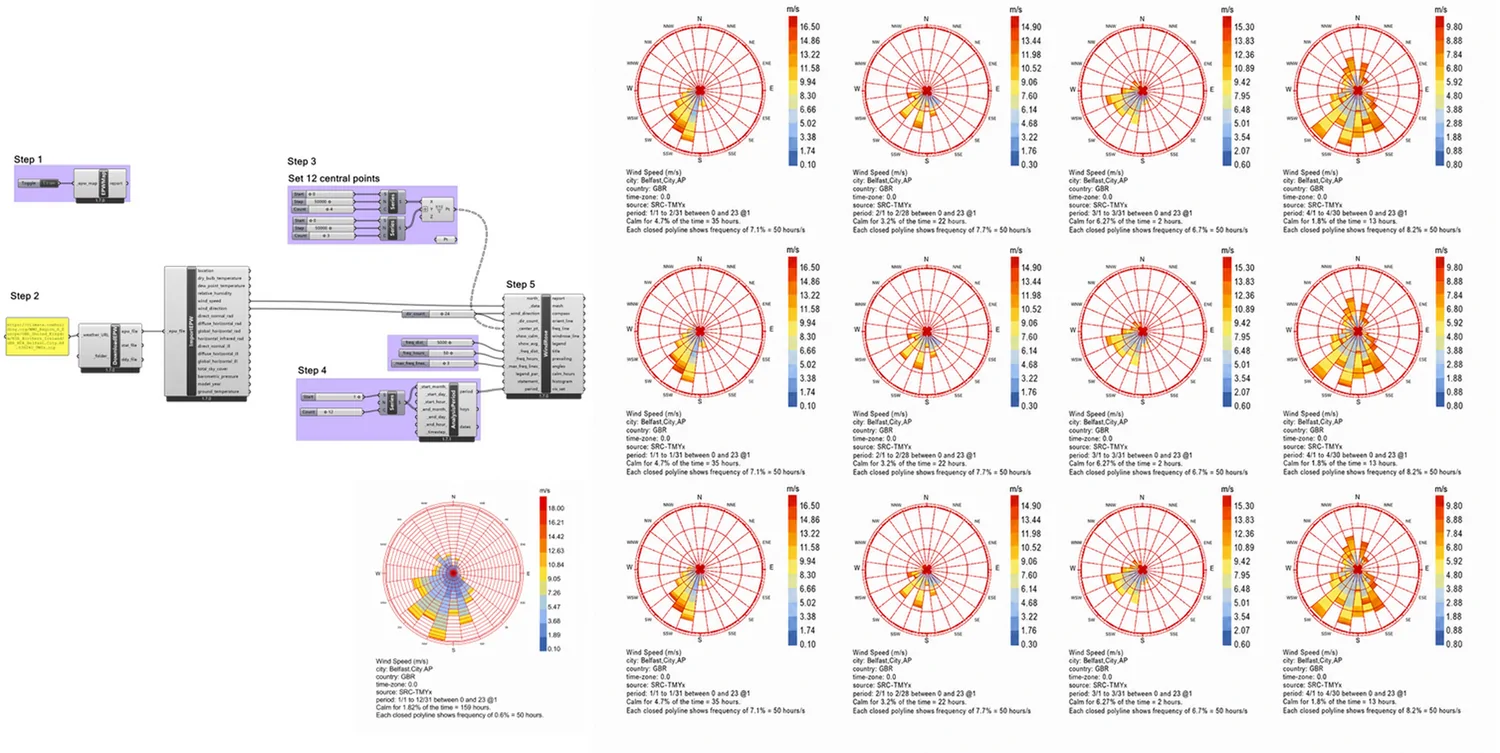
Customise weather data visualisation with wind rose analysis (author’s own)

This session developed competency in interpreting EnergyPlus Weather (EPW) files as a foundation for climate-responsive design [15]. Students first verified EPW metadata, including station location, latitude, longitude, data source, years covered, and distance to the site. Following this, they extracted core parameters, specifically dry bulb temperature, relative humidity, and wind speed and direction, to assess the data’s representativeness. Finally, students conducted annual climate and wind-rose analyses (see Figs. 3 and 4). To consolidate learning, each student created two-dimensional charts and three-dimensional visualisations of climate analysis using a case study of a typical community in Belfast. A typical Belfast community was selected as the case study site because it offered a familiar, locally relevant urban context for both cohorts, allowing students to focus on understanding the environmental situation.

#### Session 3: Daylight hours analysis and solar radiation

**Fig. 5:**
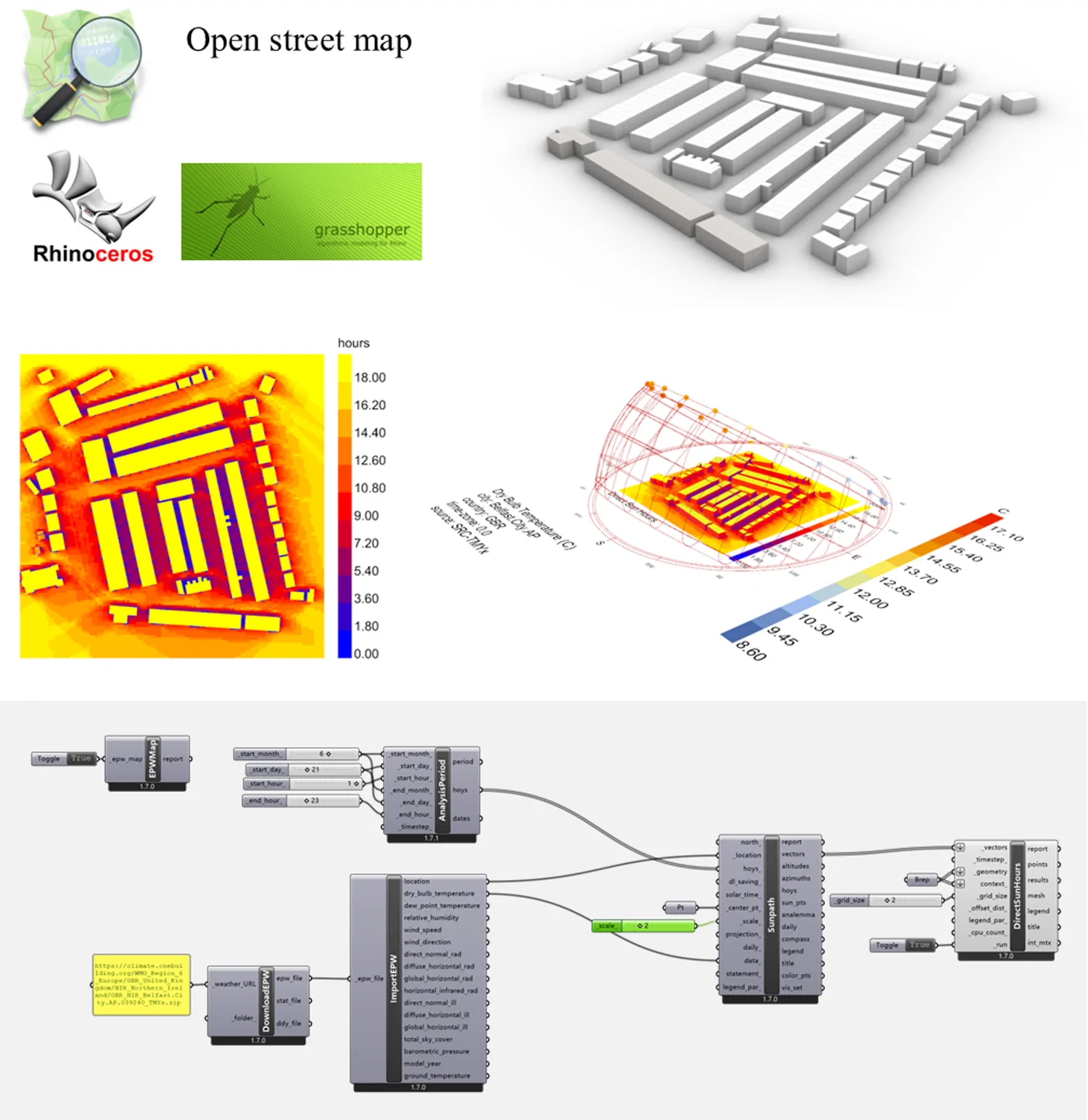
Daylight hours 3D visualization analysis (author’s own)

**Fig. 6:**
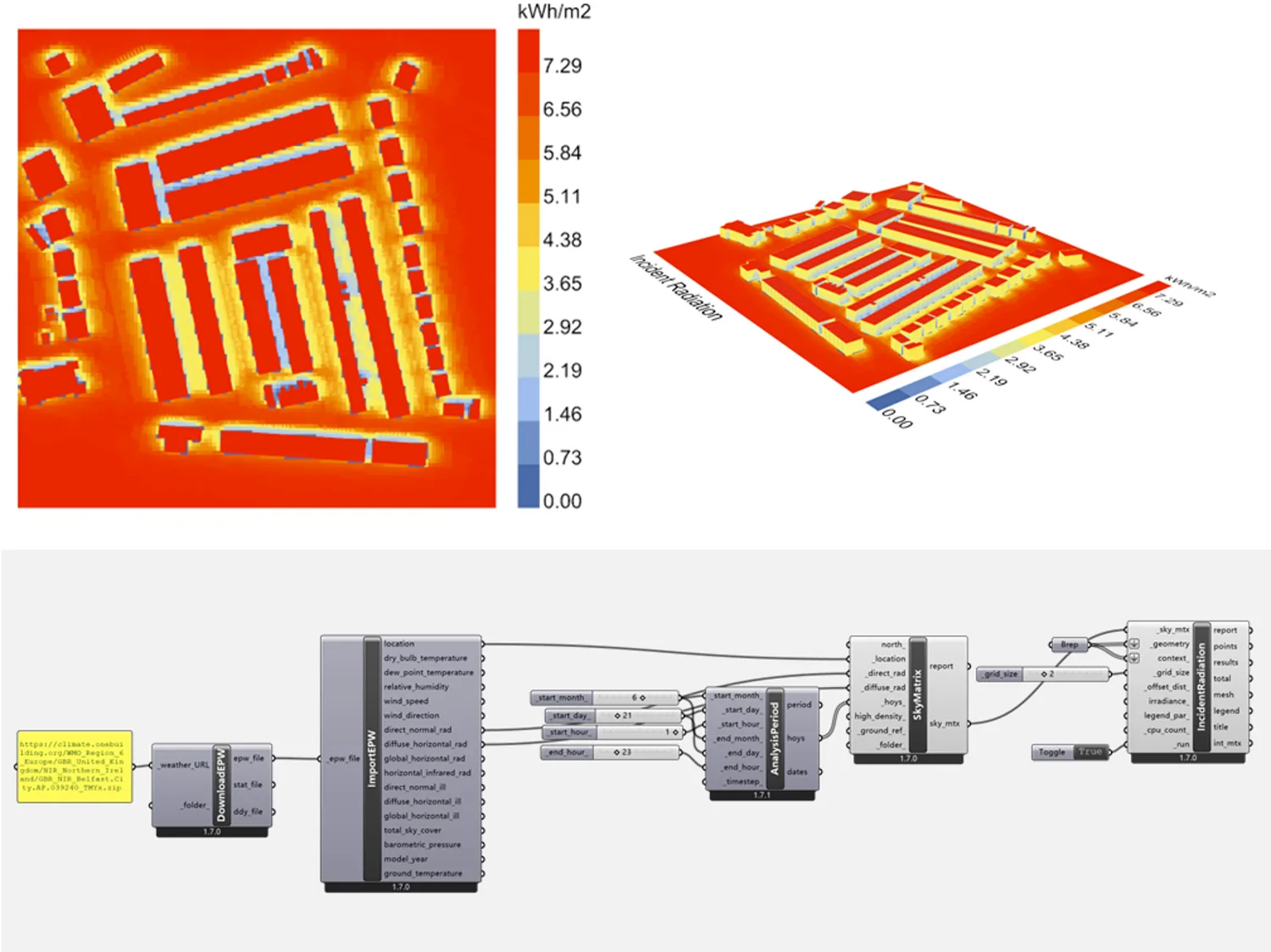
Solar radiation 3D visualisation analysis (author’s own)

Building on the EPW diagnostics from Session 2, this session examined solar exposure using Ladybug’s Sunlight Hours and Radiation components to conduct daylight-hours and cumulative solar-radiation analyses (see Figs. 5 and 6). Students first defined analysis periods (annual and season-specific windows aligned to use/occupancy) and confirmed project north and orientation scenarios. In Rhino 8, a simple 3D massing model was prepared, typically a NURBS-based block with representative façades and roof planes, sufficient to test form and orientation without detailed articulation.

Two complementary outputs were produced: (i) sunlight-hours maps (total hours of direct sun per test point over the selected period) and (ii) cumulative solar-radiation maps (kWh m²) capturing direct and diffuse components. Students generated annual plots and seasonal subsets, and ran quick sensitivity tests (e.g., ± 15° rotations, modest height changes). Common pitfalls were addressed explicitly: conflating “hours of sun” with illuminance/daylight-compliance metrics; misreading colour scales between periods; overlooking nearby obstructions; inconsistent grid densities; and omitting ground-reflectance settings.

To review learning, each student was required to generate time to analysis (e.g. 09:00, 12:00 or 18:00 on different dates). Second, each student was required to select 2–3 representative test points to extract the corresponding sunlight-hours and cumulative solar-radiation values and compare results across hours and dates using consistent units and legends.

#### Session 4: Thermal comfort analysis by universal thermal climate index

**Fig. 7:**
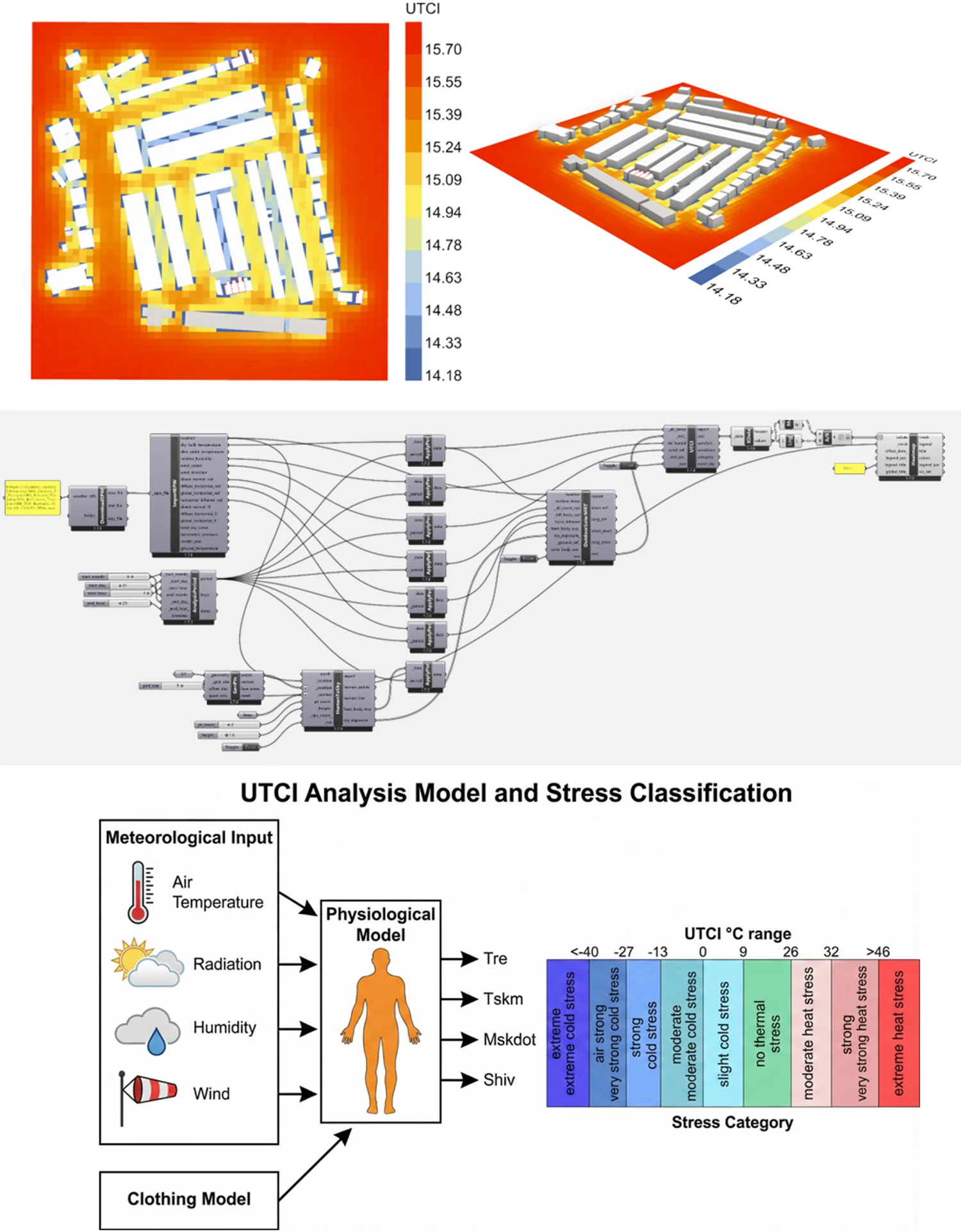
Thermal comfort analysis by universal thermal climate index (author’s own)

This session introduced outdoor thermal comfort as a core criterion for liveability in the built environment, streetscape and urban spaces, highlighting its relevance to architectural and planning design. In urban planning and architectural design, creating a comfortable thermal environment in outdoor spaces is significant in improving the attractiveness and vitality of urban outdoor spaces [30]. For architecture and planning students, comfort appraisal at the meso-scale (street, block, open-space) is a key evidence base for location, massing, and design strategies. A key focus was placed on the use of the Universal Thermal Climate Index (UTCI), introduced in 1994, which considers dry temperature, relative humidity, solar radiation, and wind speed into account and is regarded as the reference environmental temperature causing strain [25]. As an index characterising thermal comfort, different intervals of UTCI values were classified into different heat stress levels. UTCI aims to assess outdoor thermal conditions and is valid for extreme climate conditions [1, 6]. It was developed based on heat exchange theory and divided into ten categories of thermal sensation, with + 46 °C as extreme heat stress, − 46 °C as extreme cold stress, and 9 °C to 26 °C is the threshold for neutral sensation (see Fig.7) [6]. Students learned how UTCI provides more realistic measures of thermal stress than simple air temperature readings and how it can be used to assess outdoor comfort in urban environments. In session 4, using the Rhino, Grasshopper workflow with Ladybug, students produced sketch-level UTCI maps for representative hours and seasons in different locations (see Fig. 7). Inputs were standardised for reproducibility: EPW climate data and 3D models in Rhino 8. Outputs were generated for annual and seasonal windows and for time-slices (e.g., 12:00 and 15:00 on the 21st June).

#### Session 5: Review and Q&A

The final session focused on consolidating key learning outcomes and resolving any remaining technical or conceptual challenges. Students were encouraged to reflect on how the environmental simulation tools could be integrated into their ongoing or future studio projects. The open Q&A format also provided space for discussion on broader applications of simulation-based methods in sustainable urban and architectural design.

### Participants

A total of thirteen students participated in the workshops, of these eight students agree to take part in the follow up interviews (see Table 2). The cohort was evenly split by gender, with four male and four female participants. Recruitment took place through faculty networks, and all students participated voluntarily on an anonymous and informed-consent basis. The group represented diverse academic perspectives and exhibited varying levels of prior experience with digital modelling and environmental simulation tools. Based on the purpose of pilot study, sampling prioritised diversity of disciplinary background and prior digital experience rather than statistical representativeness.

**Table 2:** Prior exposure to digital modelling and environmental simulation (self-reported; not competency assessed)

Participant ID	Level	Discipline	Prior digital experience (self-reported)	Gender (optional)
P1	3rd-year undergraduate	Architecture	High	F
P2	3rd-year undergraduate	Planning	Low	M
P3	3rd-year undergraduate	Planning	Medium	M
P4	3rd-year undergraduate	Planning	Low	M
P5	3rd-year undergraduate	Planning	Low	M
P6	3rd-year undergraduate	Planning	Low	F
P7	3rd-year undergraduate	Planning	Low	F
P8	3rd-year undergraduate	Planning	Low	F

## Results

The findings presented in this section are derived from the thematic analysis of eight semi-structured interviews conducted post-workshop. Overall, the feedback indicates a strong pedagogical impact, with high positive response rates for understanding the workshop content, teaching structure, engagement with tools and sustainable design awareness. However, the data also highlights substantial friction points, with most participants recording negative or neutral responses regarding the specific challenges faced.

The following detailed analysis synthesizes these responses to answer the research questions: Sect.  4.1 examines baseline familiarity and tool engagement (addressing Q1); Sect.  4.2 investigates the specific cognitive and infrastructural barriers identified by students (addressing Q2); Sect.  4.3 explores the application of these tools in sustainable design practice (addressing Q3); Sect.  4.4 evaluates the efficacy of the pedagogical structure (addressing Q4); and Sect.  4.5 explores the students’ modes of engagement with the tools (addressing Q5).

### Evolution of digital fluency: bridging design knowledge with simulation tools

Participants’ responses to Q1 indicate a clear progression from initial unfamiliarity to a more functional understanding of environmental simulation, while also revealing variation in perceived ability and the time required for different tasks. Across the workshop, students frequently positioned themselves at the outset as having little or no prior awareness of environmental simulation, for example: “at start I had no understanding of it and I didn’t know it existed” (P2) and “before this class I didn’t know anything, environmental simulation” (P5). This novice baseline was also framed as an explicit shift in perceived knowledge gain “My understanding just went up” (P5), suggesting that the workshop played a bridging role for design and planning learners who lacked prior digital simulation fluency.

A second pattern concerns how students described moving through early conceptual opacity toward a more coherent grasp of the workflow. For some, this was articulated as overcoming an initial barrier: “once you got over the first parts of it… once you got over the basics, it was more understandable” (P6). Others described a transition from complete incomprehension to partial operational understanding through guided learning and peer observation: “initially… it was just something I just did not understand at all… but… it becomes something I’m not like entirely skilled up but it’s like a lot easier to understand I can sort of understand how things work” (P4). Notably, one participant with prior exposure to Grasshopper reported an important reframing from using it primarily for modelling to recognising it as a simulation-related workflow: “even I using the grasshopper before… I haven’t used the grasshopper as a like simulating… tool so it’s quite interesting for me” (P1).

Therefore, these accounts suggest that the workshop supported the development of an initial mental model of what simulation tools are for and how their logic operates. Crucially, several students framed their evolving understanding not merely as learning software functions but as recognising what simulation can reveal about environmental conditions. One participant described this explicitly in terms of interpretive value: “the workshop… helped develop my understanding of how that works… how the… software can actually help with figuring out different things about the environment” (P3). However, students were equally clear that increased understanding did not equate to mastery. P2 noted only reaching partial comprehension “up until last week I feel like I’ve vaguely understood how to use it a bit better but… I still need more time using it and playing around with it to understand it fully” (P2), while P4 similarly emphasised learning potential rather than full competence: “I’m not like entirely skilled up… I feel like it’s a skill I could learn in the future, if I did more work more at it”. Overall, Q1 responses characterise the intervention as enabling a transition from “zero/none” baseline awareness to early-stage functional understanding, alongside a realistic acknowledgement that sustained practice is required to stabilise independent capability.

### Barriers to effective adoption

While the workshop successfully enhanced conceptual understanding, the analysis of specific challenges (Q2) reveals substantial pedagogical friction, eliciting the highest frequency of negative or neutral responses (6 out of 8 participants). The qualitative data synthesizes these barriers into two intersecting dimensions: the epistemological challenge of grasping algorithmic logic and the logistical stratification caused by hardware inaccessibility. Several students described the cognitive load of making sense of software logic when they had “never used the software before” and “didn’t understand any of it” (P2).This difficulty was expressed in operational terms as struggling to understand how Grasshopper elements connect: “they have a like order to like, to like link each element, that’s the point I cannot understand at the first” (P1). Others framed the challenge as general technological confidence and the effort required to translate teaching content into their own coursework: “I’m not that good at, technology stuff… It was just rubbing your head around it and then, applying what you have taught us specifically to my project and my coursework, was kind of hard” (P6). Even when barriers were ultimately manageable, students still described early-stage uncertainty navigating unfamiliar interfaces—“I didn’t know where everything was so it’s hard to use at first” (P5).

Meanwhile, access and installation issues acted as a gatekeeper to active engagement, producing a practical participation gap in the classroom. Two participants explicitly reported lacking suitable portable hardware, which forced learning to become observational and deferred: “I didn’t have portable access to the software so I didn’t have a laptop… it was just learning stuff in class and then taking that knowledge back home to sort of practice at home” (P4), while another noted that working “on iPad… a lot more challenging to try and download the software or keep up with everybody… so I just… take whatever I could remember… and try and do it at home” (P3). Technical friction also carried immediate time penalties. One student described “issues with downloading” that left them “a little bit behind because everyone else was continuing on” while they were “still trying to get it downloaded” (P8). In the most constrained cases, these combined barriers translated into non-adoption in assessed work: “To be honest, I didn’t really use what we were doing in my project” (P7). Collectively, Q2 responses suggest that adoption was shaped not only by individual learning effort but also by the availability of compatible devices, smooth installation, and sufficient in-class time for synchronous practice, which determined whether students could move from observing the workflow to independently applying it.

### Application to design and planning practice

Responses to Q3 suggest a pedagogically meaningful shift in how students framed “sustainable design”: rather than treating sustainability as a purely conceptual aspiration, most participants began to describe it in relation to environmental evidence, visual outputs, and decision-support. Across the semi-structured interview, students pointed out that the studio introduced a more data-informed view of practice, including the kinds of outputs (e.g., mapped metrics, graphical representations) that they associated with “real” sustainable design work. For instance, one participant emphasised that the studio revealed the existence and complexity of specialist tools beyond presentation software, noting that these platforms can “create like graphs and maps and stuff like that” and that the workshop “made me understand how much like detail actually goes into work like this” (P3).

#### From abstract concepts to quantifiable metrics

A topic was the recognition that sustainability can be operationalised through measurable environmental parameters. One student described how the workflow foregrounded “metrics, like wind speed and different things like sunlight,” highlighting that these can be “very easily mapped to different locations,” providing “an… easy and simple to understand way of quantifying” factors that otherwise “wouldn’t be as easy,” and adding “an extra level of sustainable design… [that] wouldn’t have been available… previously” (P4). Likewise, another participant framed the learning outcome as exposure to “all those different sustainable measures” through the tools used in the studio (P8). Therefore, these accounts indicate that students were not only introduced to sustainability-related concepts, but also to the idea that sustainability claims can be supported (or challenged) through interpretive environmental evidence.

#### Disciplinary divergence in application and future design

Students also described applying simulation-informed insights in ways that reflect disciplinary priorities. The architecture participant connected the tools directly to building-scale design decisions, explaining that their “design is like a tower,” and that the workflow helped clarify “what skin I need to use for the envelope,” leading them to select “the double photon skin” to “keep the… energy” (P1). In contrast, a planning participant located the value of the tools within development appraisal and spatial configuration, describing use in “a wind farm development” and applying it to “shadow flicker,” which helped them consider “each individual turbine” so it “wouldn’t affect” surrounding conditions (P6). Another planning student similarly emphasised process-level relevance, stating that the workshops showed how the software “can be used within the planning process” to understand “sustainability of developments,” including via visualisation such as “the Sun Mops” (P2).

Beyond immediate coursework, some participants framed the studio as professional capacity building: learning such tools was described as “a good skill to have” and “great for the future,” particularly because they “didn’t know they existed before” (P7).

Importantly, not all participants could articulate an explicit link between the studio and sustainable design or co-design. One student responded directly, “I don’t know to be honest” (P5), suggesting that while the intervention enabled many learners to connect simulation outputs to sustainability reasoning, the conceptual connection was not uniformly internalised across the participants.

### Pedagogical supports and suggested improvements

Responses to Q4 highlight that students valued pedagogical supports that reduced the cognitive burden of learning unfamiliar workflows, particularly real-time guidance during sessions and the availability of materials for self-paced consolidation. Several participants explicitly appreciated in-class support and individual troubleshooting, describing that it was helpful when the instructor could “come around everyone individually and see how we’re doing” (P4). This form of situated support appeared to be especially important for students who were less confident with “technology stuff” or who were trying to translate taught steps into their own coursework context (P6).

An improvement requests concerned recording and revision, with participants noting that recorded videos enabled learning at an individual pace. One student stated that “videos are quite helpful… because you can go back and watch them” (P2), emphasising that this helped them catch up and consolidate understanding after class. Relatedly, students who faced installation or device constraints indicated that having resources to revisit was important for completing work outside the session environment (P3; P4).

A second recurring suggestion focused on pacing and synchronisation, with students emphasising the need to ensure the group reaches key milestones before moving forward. One participant recommended “making sure everyone was on the exact same page before moving on,” noting that when learners fall behind, “it’s hard to catch up” (P6). This was reinforced by comments that sessions could feel “a bit quickly” (P2) and that for some students, early confusion or lag could accumulate into disengagement, including “once I was too far behind I kind of give up” (P6).

Overall, Q4 responses suggest that students perceived the workshop’s step-by-step, demonstrative approach as broadly supportive, but they also identified two conditions that would improve equitable participation and skill consolidation: (1) recorded resources to enable self-paced replay, and (2) structured pacing checkpoints to prevent the compounding effects of early errors and reduce the likelihood of learners becoming “too far behind” (P2; P6).

### Engagement with tools

Responses to Q5 indicate a spectrum of engagement with Rhino/Grasshopper/Ladybug, shaped by both (i) the interpretive and procedural demands of component-based workflows and (ii) the extent to which students could access and practise the tools during contact hours. Across cases, engagement was strongest when students could actively manipulate the workflow and immediately see interpretable outputs, whereas engagement became constrained, sometimes displaced to after-class work, when device access, installation, or pacing limited hands-on participation.

Three distinct engagement patterns emerged from the interview data:

Evolution from hesitation to proficiency: Several students described an initial struggle followed by growing confidence. One student noted that while they “didn’t really use that much before,” they are now using Grasshopper to “analyse environment like conditions,” and even feel comfortable challenging themselves with more complex tools like Honeybee for indoor strategies (P1). Another confirmed that while the software was “difficult to understand… at the start,” it became “quite easy to understand how to get onto it fairly quickly” (P4).

Tool-Specific Utility: Engagement was highest with tools that offered immediate visual feedback. Ladybug was cited as the “easiest to use” and the most applicable for project work. One participant explicitly identified Ladybug as the most accessible entry point: “the one I find easiest to use was the ladybug one” and noted that they used it “for most of my project,” particularly because it supported “environmental impacts of my site” and helped consider “mitigation measures” (P8). This suggests that tools with clear, tangible outputs foster deeper engagement than abstract logic building.

Passive vs. Active Engagement: Hardware limitations forced some students into passive roles. One student reported that working “on iPad… a lot more challenging… to keep up with everybody,” resulting in a strategy of taking notes and attempting tasks later at home rather than practising in real time (P3). Another described lacking “portable access” because they “didn’t have a laptop,” which meant learning “stuff in class and then taking that knowledge back home… to practice at home” (P4). Finally, Q5 responses also show that when learners became significantly delayed relative to the group, engagement could collapse: “once I was too far behind I kind of give up” (P6). Taken together, these accounts indicate that tool engagement was not simply a matter of learner motivation; it was structurally conditioned by access to compatible devices, smooth onboarding, and sufficient synchronised practice time.

## Discussion

### Key findings and contributions

A key contribution of this study lies in building a bridge for environmental and climate-change knowledge, while highlighting the pedagogical value of engagement with environmental analysis tools. First, interviews indicate a consistent progression from low baseline awareness to an early, functional understanding of simulation workflows, with participants describing an increased ability to understand climate data and link outputs to design reasoning, while also acknowledging that independent mastery requires sustained practice. Second, adoption was strongly conditioned by “entry barriers”: the cognitive load of visual programming interacted with uneven hardware access and installation delays, producing a stratification between active, hands-on engagement and more passive, observational participation. Third, most participants reframed sustainability from an abstract aspiration to a set of measurables and visualisable environmental parameters (e.g., sunlight hours, solar radiation, outdoor thermal comfort).

In contribution, rather than proposing a discipline-specific tool training exercise, the study demonstrates a transferable pedagogic mechanism, rapid visual feedback coupled with scaffolded, studio-proximal tasks that can cultivate a shared “environmental language” while still enabling discipline-relevant applications at different spatial scales. Research method framed through the Future Island-Island (FII) Theory of Change. The findings also specify the enabling conditions required to mainstream such approaches, particularly the need for pre-configured digital environments, equitable computing access, and pacing checkpoints that prevent early setbacks from compounding into disengagement.

Finally, for those developing and delivering architecture and planning curricula, project lessons support investment in digital tools that can scaffold onto and within existing design-led pedagogies for studio, professional practice and technology-focused learning. Students in the study expressed an enhanced awareness of both immediate challenges for and their future practice roles in addressing climate change through responsible with design and specification. In these ways digital simulation skills arguably support teaching students (future professionals) through active processes to aid more critical selection, structuring, and application of relevant information as part of deeper, long-established goals for learning applied to the built environment (after [48, 55]).

### Applying the FII theory of change

This study provides empirical validation for the educational pathway proposed within the Future Island-Island (FII) Theory of Change, specifically confirming that targeted digital inputs (simulation tools) can successfully generate transformative outcomes (enhanced sustainability literacy) within a compressed timeframe. In response to RQ1, the findings demonstrate a pedagogical paradigm shift: the workshop transformed environmental data from a passive, regulatory obligation into an active, sustainable design driver.

The integration of Rhino, Grasshopper, and Ladybug Tools endowed students with epistemic agency, the capacity to construct knowledge through evidence rather than intuition. This aligns with the FII objective of enabling a “Green Digital Transition” by equipping future professionals with the capability to visualize invisible climate forces (e.g., solar radiation, thermal comfort). The visual and interactive nature of these tools facilitated systems-level awareness.

### Pilot study and methodological reflections

This study provides preliminary empirical support for the FII Theory of Change by showing how rapid, visually interpretable simulation outputs can shorten the distance between environmental evidence and early-stage design reasoning within a studio setting. Participants did not merely learn software steps; several described using outputs (e.g., wind-rose interpretation, solar/daylight mapping, UTCI visualisations) to justify specific “design moves” or appraisal decisions. At the same time, the pilot indicates that such agency is contingent on implementation conditions, particularly equitable device access and reduced installation friction, without which engagement shifts from active experimentation to delayed, after-class replication.

### Limitation and future work

In this study, there also presents several limitations. First, the sample size was small (*N* = 8) because participation in the class was voluntary rather than mandatory. The pilot was delivered during a busy academic period with heavy coursework commitments, which constrained the number of students able to take part. While the diverse academic backgrounds of participants enriched the data, the cohort’s voluntary participation may have introduced self-selection bias, favouring students already motivated to engage with digital tools. Second, the evaluation relied on self-reported perceptions rather than objective performance assessments (e.g., pre–post tests of simulation, rubric-based grading of outputs, or log-based indicators of tool use). While these reflective accounts are well-suited to examining perceived learning, confidence, and sense-making processes in a short studio-embedded pilot, they may be susceptible to social desirability and recall bias and cannot, on their own, quantify the magnitude of skill acquisition or task competence. Future iterations can consider triangulating self-reports with standardised marking criteria for the design project to strengthen validity and support more generalisable claims. Finally, because this was a short, studio-embedded pilot, we did not collect delayed follow-up evidence (e.g., subsequent project artefacts, rubric-based portfolio assessment, or delayed post-tests); as such, the study cannot speak to longer-term retention or later project uptake. Future work should therefore adopt a longitudinal design to examine whether the observed short-term gains persist and translate into measurable changes in later studio outputs and/or professional practice.

Future research should address these limitations by testing the FII framework across multiple institutions and expanding the sample size to validate its scalability. Longitudinal studies are needed to track how these initial digital skills evolve into professional practice over time. Furthermore, future research should employ a hybrid approach design, combining qualitative affective data with objective indicators such as the technical quality of planning assessments or the performance logic of studio design works, thereby providing strong evidence for the impact of pedagogical methods on practical application. Given the considerable advances and use of GenAI within education as well as practice, it would be beneficial for further studies to investigate whether GenAI-supported approaches can assist students in interpreting simulation outputs, communicating environmental-performance evidence and receiving adaptive learning support, while critically evaluating their accuracy, transparency and potential risks.

## Conclusion

This study provides empirical evidence for a scaffolded, interdisciplinary approach to mainstreaming environmental simulation within builtenvironment education. Anchored in the FII Theory of Change, the pilot indicates that a workshop embedded pathway, centred on Rhino, Grasshopper, and Ladybug Tools. Despite the compressed delivery period, participants progressed from limited digital proficiency to demonstrable operational capability, applying evidence-based performance metrics to inform architectural and urban design decision-making.

At the same time, the pilot surfaced structural inequities in learning conditions across Northern Ireland, particularly differences in baseline cognitive load and the additional burdens introduced by uneven hardware access and computing capacity. These disparities shaped students’ pace of progress and their ability to sustain iterative simulation design cycles, underscoring that pedagogical effectiveness is inseparable from infrastructural readiness.

Accordingly, this research cannot be achieved through software provision alone; it requires a systemic reconfiguration of institutional support. Targeted friction-reduction measures, such as equitable access to high-performance computing, proactive technical onboarding, and differentiated learning supports, are essential to democratise participation in simulation-enabled design. With these conditions in place, institutions can move beyond imparting sustainability concepts toward cultivating genuine digital agency, enabling future practitioners to translate environmental evidence into actionable, responsible design outcomes.

## Supplementary Information

Below is the link to the electronic supplementary material.

Supplementary Material 1

Supplementary Material 2

Supplementary Material 3

Supplementary Material 4

Supplementary Material 5

Supplementary Material 6

Supplementary Material 7

Supplementary Material 8

## Data Availability

The datasets generated during and/or analysed during the current study are not publicly available due to ethical restrictions and the necessity to preserve participant anonymity but are available from the corresponding author on reasonable request.

## Abbreviations

APC: Assessment of Professional Competence
BIM: Building Information Modelling
CFD: Computational fluid dynamics
CPD: Continuing Professional Development
CSR: Corporate social responsibility
EPW: EnergyPlus Weather
FII: Future Island Island
GIS: Geographic Information Systems
QUB: Queen’s University Belfast
RTPI: Royal Town Planning Institute
SD: Sustainable Development
SDGs: Sustainable Development Goals
TCPA: Town and Country Planning Association
UTCI: Universal Thermal Climate Index
