# Supplementary material for: A pilot study integrating environmental simulation for climate responsive architecture and planning curricula
Source: Discov Educ. 2026 Nov 14;5(1):1335. doi: 10.1007/s44217-026-02391-z (PMC13769756; doi:10.1007/s44217-026-02391-z)
Supplement: Supplementary file 1 [file 44217_2026_2391_MOESM8_ESM.docx]

**QUB_P1**

**How has your understanding of environmental simulation evolved during the workshop?**

Answer from QUB: well, actually first time to like hear about it and then even I using the grasshopper before, but like isn't really for the like the modeling of the like building only it's not I didn't really have.. I haven't used the grasshopper as a like simulating, the tool so it's quite interesting for me, so that's why I'm joined this workshop and then I think it's quite useful to like analyze the environmental like environment environment system something like that. and then, uh I'll say it's kind of hard to like use it for the some like accurate um it's kind of hard to use it for the accurate site for the building cause only have a few point in the reason I spill, even like in the Belfast, they have only 2 spot, like 2 point for the like the information they got, so yeah but like I think it's generally it's kind of easy to use for analyze.

**What were the main challenges you faced while learning and applying environmental simulation in your project?**

uh main challenge, uh for me it's kind of at the first time, it's first time to use that simulation, so like I need to understand what is like the component you know the they have a like order to like, to like link each element, that's the point I cannot understand at the first, but like I think it's quite, okay for me to learn. But like I feel if someone want to try to learn who like don't ever use the grasshopper before, I think it's kind of struggle point to like understand what is this program is basically.

**How did this studio shape your understanding of sustainable design and co-design processes?**

actually I using that program after the workshop to apply the my site analysis, like in my 3rd year project and then.. Um, personally, the design of my I mean my design is like a tower, so it's kind of easy to understand like what skin I need to use for the envelope, like of the building. So I choose the double photon skin to like keep the dormer energy and something. yeah so it's really, I would say that it's not really accurate, it's generally can understand like, what I need to do for, like keep the energy or like save the like material or something like that.

**How did the workshop structure, assignments, or teaching methods impact your learning experience? Or Were there any teaching practices that you found particularly effective or that could be improved?**

I think it's all good for me and you already like show the like your screen on it like beam projector and then I can see the what you press for the and then for the element and how you link together. I think it's kind of easy to understand for me, and then you also like put the like in um, like describe what is, what is that for also, so I think it's like generally I feel like, it's really good experience to learn I guess and then, it's it would be better like if you give it like more assignment. I think like to practice more,

**How did you engage with the environment simulation tools (e.g. Rhino, Grasshopper, Ladybug Tools) thought the workshop?**

so I think I feel like, I'm getting better to like using the grasshopper, and then like ladybug tools, after like workshop, and even the grasshopper, I didn't really use that much before, but like nowadays I using grasshopper to like have a like um, to analyze environment like conditions and everything, and I try to, I'm more feel comfortable to like challenge to like honeybee for the in indoor, yeah strategy something I analyze something, I think like I feel free to like try another tools, linked with the grasshopper, so it's good to, learn I feel yeah..

**Ulster_P2**

**How has your understanding of environmental simulation evolved during the workshop?**

Well, at start I had no understanding of it and I didn't know it existed and up until last week I feel like I've vaguely understood how to use it a bit better but I feel like I still need more time using it and playing around with it to understand it fully and actually how to use it.

**What were the main challenges you faced while learning and applying environmental simulation in your project?**

I would say the main challenges again probably because I'd never used the software before, I didn't understand any of it but by going through the workshops week by week, and watching your tutorials it, helped me gain a better understanding of how to use it, and put the parts together within rhino,

**How did this studio shape your understanding of sustainable design and co-design processes?**

Your workshops helped show how this software is used within or can be used within the planning process and how to use the software to understand more sustainable processes and sustainability of developments with like the Sun Mops. I can be implemented in in the work,

**How did the workshop structure, assignments, or teaching methods impact your learning experience? Or Were there any teaching practices that you found particularly effective or that could be improved?**

uh I find it helpful watching you, but I feel at times it was a bit rushed not well not rushed but like done a bit quickly, for us to fully understand it how you got to each part and through each part, because it is something new that we're trying to learn, but again the videos that you uploaded and shared were quite helpful to have so that we could also go back and look through those and watch them to gain a better understanding

**How did you engage with the environment simulation tools (e.g. Rhino, Grasshopper, Ladybug Tools) thought the workshop?**

Well, I followed your instructions and downloaded all of them which at times was a bit long and hard to download but I got there the software's were quite difficult to understand how to get onto grasshopper in rhino at the start. But then it's quite easy to understand how to get onto it fairly quickly

**Ulster_P3**

**How has your understanding of environmental simulation evolved during the workshop?**

in general, the workshop, I got find out more about how the like software can actually help with figuring out different things about the environment it's just like the those of this like a some patterns or something like that there so the workshops that has helped develop my understanding of how that works.

**What were the main challenges you faced while learning and applying environmental simulation in your project?**

My software of my way to do it anywhere was on iPad so unlike everyone else on a laptop it was a lot more challenging to try and download the software or keep up with everybody because it was couldn't really do it in here so I just want to just take whatever I could remember right down and try and do it at home on the computer at home so it that's probably the main challenge I faced.

**How did this studio shape your understanding of sustainable design and co-design processes?**

Maybe sort of understand there's a lot more going into the design like could it just been like instead of just like Powerpoint or something like that like there's these different softwares like they have a very complex and so whenever you figure out what to do in those different software then they can actually create like graphs and maps and stuff like that so you know this here like workshop, it's really made me understand how much like detail actually goes into work like this

**How did the workshop structure, assignments, or teaching methods impact your learning experience? Or Were there any teaching practices that you found particularly effective or that could be improved?**

I like the fact that you went wide, everybody just to make sure they were trying to stay in the same like way of length as you cause they just kept going on and on and everybody would have just been completely lost so that was good but maybe improve and maybe just maybe had like a few more steps to make it, a bit easier to grasp if that makes sense for people because a lot of people a bit confused by how it worked but other than that it was in fine, some more you could have done.

**How did you engage with the environment simulation tools (e.g. Rhino, Grasshopper, Ladybug Tools) thought the workshop?**

I found it quite difficult because I was trying to do it on an iPad as opposed to a laptop but I sort of just watched other people do and was listening along to audio steps so we can try to do it at home but sort of hard to engage for me just because I couldn't physically do it myself but it seemed like if I had like an opportunity to do it myself on an actual laptop it would have been a lot easier to like work through but then this was a divine of grasp up a ladybug all in having to like download each of them and all of them work collectively. I think that's quite cool!

**Ulster_P4**

**How has your understanding of environmental simulation evolved during the workshop?**

Well, initially it was something I'd like when I initially looked at, it was just something I just did not understand at all. but just through it lessons and through your guidance and from watching other people do it as well it becomes something I'm not like entirely skilled up but it's like a lot easier to understand I can sort of understand how things work and I feel like it's a skill I could learn in the future, if I did more work more at it

**What were the main challenges you faced while learning and applying environmental simulation in your project?**

Well, the main challenge I face is I didn't have portable access to the software so I didn't have a laptop I was doing most of the work on a desktop PC at home so it was just learning stuff in class and then taking that knowledge back home to sort of practice at home. So I was just doing all the things that listen to you watching videos on it, then watching my friends do as well, just taking as much information I could while I was here, but I feel like I would have just had maybe a greater understanding of it. if I did have access to a laptop or a school or a university computer that did have access to the software.

**How did this studio shape your understanding of sustainable design and co-design processes?**

It helped me understand that there's a lot of metrics, like wind speed and different things like sunlight not that they can be very easily mapped to different locations, and provides just a very like easy and simple to understand way of quantifying a lot of those factors that maybe wouldn't be as easy to and it just gives an extra level of sustainable design that just maybe wouldn't have been available and previously.

**How did the workshop structure, assignments, or teaching methods impact your learning experience? Or Were there any teaching practices that you found particularly effective or that could be improved?**

I feel like most of your demonstrations were very well. Explained and then come around everyone individually after I think that was very helpful as well. As for improvements I can't think of any offhand so I think that's all good. I think the sessions were very well very well done

**How did you engage with the environment simulation tools (e.g. Rhino, Grasshopper, Ladybug Tools) thought the workshop?**

Well, obviously I didn't engage much in class, apart from just working with like friends and watching them do it. But at home, I was mainly just taking the information from the workshops and then putting that into trying to best replicate stuff at home.

**Ulster_P5**

**How has your understanding of environmental simulation evolved during the workshop?**

before this class I didn't know anything, environmental simulation so after taking all these workshops. My understanding just went up

**What were the main challenges you faced while learning and applying environmental simulation in your project?**

It was a new program for me and I didn't quite understand all the it was this new program and I didn't understand why. At first we had to use it but after 10 a few of the workshops, as I realized then, and the laptop I was using wasn't quite, a laptop I was using.. I didn't know where everything was so it's hard to use at first.

**How did this studio shape your understanding of sustainable design and co-design processes?**

I don't know to be honest. It's just, yeah that's all I don't know.

**How did the workshop structure, assignments, or teaching methods impact your learning experience? Or Were there any teaching practices that you found particularly effective or that could be improved?**

I learn by watching things, and like, I'm a visual learner, the only thing I would like preferred like preferred for the lessons to be recorded. So I got watch back and whatnot but not like, it was all good although not,

**How did you engage with the environment simulation tools (e.g. Rhino, Grasshopper, Ladybug Tools) thought the workshop?**

It was fun learning about all the programs I haven't used before and I haven't heard of before, and I was unsure of what the, I was unsure of the I was unsure of the purpose of them but yeah I got to know them and understand. I enjoyed working with them.

**Ulster_P6**

**How has your understanding of environmental simulation evolved during the workshop?**

I didn't really have any understanding of like the simulations or how to do them, so I now have more of an idea of them. It was I found it kind of difficult but um, once you got over the first parts of it, then it was fine, once you got over the basics, it was more understandable.

**What were the main challenges you faced while learning and applying environmental simulation in your project?**

I'm not that good at, technology stuff anyway that sort of thing so it was probably that and just because it was completely new to me. It was just rubbing your head around it and then, applying what you have taught us specifically to my project and my coursework, was kind of hard but figured it out and got right up.

**How did this studio shape your understanding of sustainable design and co-design processes?**

Well, in my coursework it was kind of I was doing like a wind farm development and I tried to use it for shadow flicker which is quite important with that with all sort of development. So it helped me understood like what apart each individual turbine, so it wouldn't affect how to hang around it.

**How did the workshop structure, assignments, or teaching methods impact your learning experience? Or Were there any teaching practices that you found particularly effective or that could be improved?**

um it was just something different, and it was more like focused like we've kind of had like had to do stuff on GIS. but it hasn't been very specific to like us, it's always been like different courses, and there are courses more focused on like the technology side of things but because it was just us it was kind of more useful. and easy to follow.

**How did you engage with the environment simulation tools (e.g. Rhino, Grasshopper, Ladybug Tools) thought the workshop?**

It was all quite challenging at the start, but again and downloading the stuff was quite challenging, but it was alright once you got it downloaded and got the basics of it. I do feel like I have more of an understanding of what rhino and grasshopper is but I probably do with more focusing on it to perfect.

**Ulster_P7**

**How has your understanding of environmental simulation evolved during the workshop?**

well I started a no understanding basically of this, so I've gained a bit of knowledge. But I'm not very good at computers, and stuff so it was a bit hard but I've definitely Learned some, because I really went from zero to a good bit of knowledge, So it has evolved a good but yeah,

**What were the main challenges you faced while learning and applying environmental simulation in your project?**

To be honest I didn't really use, what we were doing in my project.

**How did this studio shape your understanding of sustainable design and co-design processes?**

Well it was good to learn how to do it, I think that's a good skill to have. yeah I just I think it will be great for the future to know how to use like tools like that. Like, I don't know they existed before like ladybug running, so um it has developed my understanding, and I think it'll be good. It'll be a good skill to have.

**How did the workshop structure, assignments, or teaching methods impact your learning experience? Or Were there any teaching practices that you found particularly effective or that could be improved?**

I think it was a bit hard. I think me personally I was falling behind in class like there was a girl she was ahead of everyone. It seemed like she knew what she was doing, and I'm quite bad at using computers, but so I was kind of fallen behind I think it would have been better if it was step by step in front of the whole class instead of like going around individually. I know that kind of helped going around it kind of helped, people catch up with it but I think if we more so like made sure that everyone was on the exact same page before moving on to the next step. So we were all like on the same, part of it at the same time would've been better, because yeah I know I'm not the only one that fell behind. I think a lot of people were a good bit behind and then once you're behind. It's hard to catch up again.

**How did you engage with the environment simulation tools (e.g. Rhino, Grasshopper, Ladybug Tools) thought the workshop?**

I find out quite hard. I don't know if it was just me, I like I think other people might have find it okay but um I find it quite difficult to use so I think, I was kind of struggling with engagement and all yeah…I said a class I never really used it, and apart from trying to catch up, maybe the first few times, but once I was too far behind I kind of give up. and I do still have a download at all, but it was hard even to download as well. Now I definitely think I'll use that in the future, it's a good thing for like a job I like to put on to like learn how to use.

**Ulster_P8**

**How has your understanding of environmental simulation evolved during the workshop?**

well I've never used it before so starting from not ever using that before. Yeah it was helpful for this module particularly for the environmental impact assessment because I did a solar farm on the likes of like Ladybug and stuff that showed like climate and um solar radiation which was helpful for my doing solar farm.

**What were the main challenges you faced while learning and applying environmental simulation in your project?**

yeah so since I'd never used it before and obviously we had the issue with downloading the both of us. and so that was a challenge I faced at the start and sort of got me to be a little bit behind because everyone else continuing on method and I was still trying to get it downloaded, which was hard it was but then once I had got up I helped me with like my learning towards all the different environmental impacts. and the tools that you could use on it were helpful,

**How did this studio shape your understanding of sustainable design and co-design processes?**

um yeah it was good for learning about sustainable, design because the tools helped you with learning all those different sustainable measures

**How did the workshop structure, assignments, or teaching methods impact your learning experience? Or Were there any teaching practices that you found particularly effective or that could be improved?**

yeah so I would have never known how to get most of the stuff that I needed for my assignment. and that tool was able to sort of help me with all the different things that provide it like the things I'd mentioned earlier. and solar radiation wind and climate and temperatures and stuff like that

**How did you engage with the environment simulation tools (e.g. Rhino, Grasshopper, Ladybug Tools) thought the workshop?**

and the one I find most easiest to use was the ladybug one that was probably the one that I used for most of my project. just because I had like a lot of things to help me measure for like the impacts and then of environmental impacts of my site in the area and then I was it helped me sort of just go from there and think of ways to come up with my egression measures
